# Allergenicity assessment of GMOs by the European safety authority

**DOI:** 10.1186/2045-7022-1-S1-S12

**Published:** 2011-08-12

**Authors:** Jean Michel Wal

**Affiliations:** 1 Service de Pharmacologie et Immunologie (SPI), Laboratoire d'Immuno-Allergie Alimentaire, Gif sur Yvette cedex, France

## 

The risk of genetically modified organisms (GMOs) and foods derived from GMOs for human health and particularly their allergenicity must be assessed before they are authorized to be put on the market in the European Union. Data from the studies performed by the Applicant are evaluated by the GMO Panel of the European Food Safety Authority (EFSA). It then publishes a scientific opinion including a conclusion on the food safety for the information of the Risk Manager (i.e. the European Commission).

Recommendations for allergenicity assessment have been provided by international scientific committees and particularly by EFSA in recent reports. Both the risk of *de novo* sensitization of genetically predisposed consumers and of elicitation of an allergic reaction in consumers already sensitized (e.g. to cross reacting allergens) must be assessed on a case by case basis. Two issues must be addressed :

i) The allergenicity of the newly expressed protein(s) encoded by the transgene inserted in the GMO. The assessment is based on the so called weight of evidence approach because no characteristics can allow to definitely predict the (absence of) allergenicity of a protein. Information of different nature including origin, structure, immunological and physico chemical properties of the protein, obtained using *in silico*, *in vitro* and possibly *in vivo* tests are considered to conclude on the likelihood of allergenicity.

ii) The allergenicity of the whole GM food. Applicant must test that no unintended effect of the genetic modification, e.g. resulting in an over-expression of endogeneous allergen(s), has occurred by comparing the allergen repertoire of the GMO and its non GM counterpart. This may be performed by profiling methodologies using *–omics* technologies, generally in association with the use of allergic human sera as probes.

For both cases, scientific grounds for the design and interpretation of strategies and testing methods that may be used on a case by case basis will be discussed in the presentation.

